# Microglia Function on Precursor Cells in the Adult Hippocampus and Their Responsiveness to Serotonin Signaling

**DOI:** 10.3389/fcell.2021.665739

**Published:** 2021-05-24

**Authors:** Andrei Turkin, Oksana Tuchina, Friederike Klempin

**Affiliations:** ^1^School of Life Sciences, Immanuel Kant Baltic Federal University, Kaliningrad, Russia; ^2^Department of Psychiatry and Psychotherapy, Charité University Medicine Berlin, Berlin, Germany

**Keywords:** microglia, serotonin, hippocampus, BDNF, fluoxetine, neuroinflammation

## Abstract

Microglia are the resident immune cells of the adult brain that become activated in response to pathogen- or damage-associated stimuli. The acute inflammatory response to injury, stress, or infection comprises the release of cytokines and phagocytosis of damaged cells. Accumulating evidence indicates chronic microglia-mediated inflammation in diseases of the central nervous system, most notably neurodegenerative disorders, that is associated with disease progression. To understand microglia function in pathology, knowledge of microglia communication with their surroundings during normal state and the release of neurotrophins and growth factors in order to maintain homeostasis of neural circuits is of importance. Recent evidence shows that microglia interact with serotonin, the neurotransmitter crucially involved in adult neurogenesis, and known for its role in antidepressant action. In this chapter, we illustrate how microglia contribute to neuroplasticity of the hippocampus and interact with local factors, e.g., BDNF, and external stimuli that promote neurogenesis. We summarize the recent findings on the role of various receptors in microglia-mediated neurotransmission and particularly focus on microglia’s response to serotonin signaling. We review microglia function in neuroinflammation and neurodegeneration and discuss their novel role in antidepressant mechanisms. This synopsis sheds light on microglia in healthy brain and pathology that involves serotonin and may be a potential therapeutic model by which microglia play a crucial role in the maintenance of mood.

## Introduction

In the adult brain, microglia are the resident macrophages and, as such, a unique cell population interacting with neurons, astrocytes, oligodendrocytes, and the various signaling molecules. Characterized by Iba-1 and CD11b immunoreactivity (expressed in resting and activated cells; Franco and Fernández-Suárez, 2015), microglia exhibit a diverse, dynamic morphology that allows a quick response to changes in the environment. Under physiological conditions, highly branched microglial cells constantly sense the environment to maintain homeostasis, modulate synapse maturation and connectivity, and regulate neuronal activity (Kettenmann et al., 2011). In the hippocampus, in particular, microglia display a vigilant phenotype (Grabert et al., 2016); they take part in learning-dependent synaptic plasticity and neural network excitability, and release of growth factors and neurotrophins, e.g., brain-derived neurotrophic factor (BDNF) (Parkhurst et al., 2013), involved in memory formation. As part of the limbic system, the hippocampus plays a central role in learning, especially in the encoding and retrieval of episodic and spatial memories (Buzsaki and Moser, 2013). Importantly, microglia contribute to the lifelong generation of new neurons in the hippocampus. Set in the dentate gyrus, neural stem cells (NSCs) retain fate plasticity and respond to a variety of local cues and extrinsic stimuli that foster a neuronal fate. Most of the newly generated cells die before maturation into granule neurons (Dayer et al., 2003) as a strategy balancing cell proliferation vs. cell death. In their role in phagocytosis of damaged cells and debris, recent studies attribute non-activated microglia to the control over the neuronal cell pool by removal of apoptotic progenitor cells (Sierra et al., 2010).

A prominent local component of the neurogenic niche is serotonin (5-HT). Modulating both proliferation and survival of newly generated cells, serotonin is a key regulator of adult neurogenesis (Alenina and Klempin, 2015) and, together with BDNF, is involved in antidepressant mechanisms (Mattson, 2008; Molendijk et al., 2011; Kronenberg et al., 2018a). Accumulating evidence from rodent studies and *in vitro* modeling indicates that microglia interact with local hormones and neurotransmitters by the expression of various receptors (Pocock and Kettenmann, 2007). Among them are metabotropic glutamate receptors, the chemokine fractalkine receptor (CX3CR1) (Sellner et al., 2016), and various serotonin receptor subtypes, particularly 5-HT2B (Krabbe et al., 2012). Expressed on microglia subpopulations (Kettenmann et al., 2011), receptors’ attraction to neuronal secretion of signaling molecules assists surveillance of the microenvironment (Szepesi et al., 2018).

Upon stimulation, microglia become activated, proliferate, lose their ramified morphology, and display the first innate immune defense (Beynon and Walker, 2012). They rapidly act by secretion of distinctive inflammatory cytokines, e.g., interleukins (ILs), interferons (IFNs), and tumor necrosis factors (TNFs) that in turn modulate the release of neurotransmitters and neurotrophins. Depending on the microenvironment, cytokines function pro- (i.e., IL-1β, IL-6, IL-18, and TNF-α) or anti-inflammatory (i.e., IL-4 and IL-10) (Suzumura, 2013; Franco and Fernández-Suárez, 2015). BDNF exerts primarily anti-inflammatory and neuroprotective effects (Chen et al., 2016). Dysregulation of the immune defense function leads to neuroinflammation and neuronal cell death. Excessive glutamate release is particularly neurotoxic (Lewerenz and Maher, 2015). Microglia-mediated “neuroinflammation” is increasingly recognized to contribute to the development and progression of neurodegenerative diseases and psychiatric disorders. Structural changes in neuroplasticity, altered intrinsic signaling, i.e., of serotonin and BDNF, and impaired neurogenesis are observed in stress-related events, Alzheimer’s disease (AD), or major depression. This review will summarize microglia function on precursor cells in the adult hippocampus, their contribution to neuroplasticity, and modulation by physiologic stimuli. We will synopsize how their behavior is altered upon activation leading to neurodegeneration and will discuss microglia response to serotonin signaling and 5-HT receptor function on microglial cells *in vitro* and *in vivo.* We will complete by describing the role of microglia in serotonin-mediated antidepressant action, e.g., in response to the selective serotonin reuptake inhibitor (SSRI) fluoxetine. Key findings are summarized in Table 1.

**TABLE 1:** Summary of recent findings on microglia function in neuroplasticity and neuro-inflammation in the hippocampus, with focus on serotonin and antidepressant action.

**Microglia in neuroplasticity and neuroinflammation**		
Kettenmann et al., 2011; Grabert et al., 2016		Microglia represent a diverse and vigilant phenotype with high numbers in the dentate gyrus
Pocock and Kettenmann, 2007; Szepesi et al., 2018	5-HT, 5-HTR	Microglia interact with local neurotransmitters and hormones
Gemma and Bachstetter, 2013		Microglia contribute to adult neurogenesis
Pascual et al., 2012	TNF-α, ATP, glutamate	Synapse plasticity *via* microglial release of TNF-α and ATP triggering nearby astrocytes to release glutamate
Trang et al., 2011	ATP, BDNF	ATP-P2X4 drives BDNF release from microglia
Parkhurst et al., 2013	BDNF	Microglia-mediated synaptogenesis *via* BDNF
Ferrini and De Koninck, 2013	BDNF	In neuroinflammation, microglia-mediated BDNF signaling causes synaptic disinhibition
Sierra et al., 2010; Diaz-Aparicio et al., 2020		Microglia phagocytosis of apoptotic newborn cells in the dentate gyrus through the phagocytosis secretome
Klempin et al., 2013	Tph2, Iba-1	Running-induced microgliosis in wild-type hippocampus that is further enhanced in mice lacking brain serotonin
Wasinski et al., 2018	B2R, Iba-1	Running-induced microgliosis in hippocampus of bradykinin B2 receptor knockout mice
Ehninger and Kempermann, 2003; Ehninger et al., 2011	Iba-1	Physical exercise increases newborn microglia numbers in cortex, but decreases the amount in adult amygdala
Ali et al., 2019	Iba-1	Long-term ENR enhances microgliosis in adult hippocampus and amygdala, hypertrophied and ramified microglia morphology
de Sousa et al., 2015; de Oliveira et al., 2020	Iba-1	Increased microglia complexity in CA3, reduced diversity in molecular layer in ENR
Johnson et al., 2003; Szuhany et al., 2015	BDNF	Physical exercise strongly induces BDNF release in rodents, and humans
Moon et al., 2016	BDNF	Skeletal muscle releases cathepsin B during running in monkeys that affects BDNF levels in the brain
Tuchina et al., 2018		Interplay of the endocrine, immune and limbic systems during stress
Goronzy and Weyand, 2013		Senescent myeloid cells decrease process motility and chemotaxis
Pickering and O’Connor, 2007	TNF-α, IL-1, IL-18 in AD	Enhanced release of pro-inflammatory cytokines in disease progression
Shen et al., 2018	AD	Dysfunctional microglia in disease progression
Ng et al., 2018	IL-1β, IL-6 in AD, major depression	Enhanced peripheral levels in patients
Burbach et al., 2004	BDNF in AD	In AD inflammation, release of BDNF by microglia in close proximity to plaques
Floden et al., 2005	TNF-α, glutamate in AD	b-amyloid-induced microglia-mediated cell death *via* the release of TNF-α and glutamate
Makar et al., 2009	BDNF, IL-10	BDNF promotes IL-10 release in multiple sclerosis
Borsini et al., 2015	Cytokines	Distinctive cytokines acting on cell proliferation and differentiation *in vitro*
Kelly et al., 2001; Lim et al., 2013	IL-10	Anti-inflammatory; promotes synaptic plasticity and long-term potentiation
Cacci et al., 2008; Willis et al., 2020	IL-10 IL-6	Potent suppression of pro-inflammation and robust support of adult neurogenesis
Paolicelli et al., 2011; Sellner et al., 2016; Bolós et al., 2018	Fractalkine/CX3CR1 *Cx3cr1*	Prominent chemokine regulator of neuron–microglia communication in the postnatal and adult dentate gyrus; important for synaptic pruning
Bachstetter et al., 2015; Milior et al., 2016	Fractalkine/CX3CR1 in AD, chronic stress	Deficiency results in microglia-induced pro-inflammation and impaired neurogenesis
Monje et al., 2003; Bastos et al., 2008; Fujioka and Akema, 2010	LPS, BrdU and neuronal markers	Dose- and time-dependent effects on cell proliferation, survival and neuronal fate in the adult dentate gyrus, *in vivo/in vitro*
Ekdahl et al., 2003	LPS, BrdU	Negative correlation of activated microglia-newborn cells
Mizoguchi et al., 2014 Zhang et al., 2014 Wu et al., 2020	LPS BDNF-TrkB	(LPS-induced) microglia activation, transformation can be reduced by BDNF or TrkB agonist treatment; BDNF sustains Ca2^+^ elevation

**Serotonin–Microglia function**		

Stagaard et al., 1987; Vetreno et al., 2017	5-HT, Tph2, VMAT, SERT, Iba-1, CD11b	Serotonin depletion increases microgliosis in dorsal raphe, and subcommissural organ
Krishna et al., 2016	LPS, 5-HT	Transient increased microglia numbers and a depressive-like phenotype upon chronic LPS
Carabelli et al., 2020	LPS, Omega-3, 5-HT	Fish oil reverses depression-like behavior, increases serotonin in the hippocampus
Albertini et al., 2020	5-HT	Microglial processes in close proximity to serotonergic axons in the adult hippocampus
Seifert et al., 2011	5-HT, Ca2^+^	Transient enhanced Ca2^+^ signaling in response to serotonin *in vitro*
Glebov et al., 2015	5-HT2A/B, 5-HT4, Ca2^+^	Serotonin stimulates secretion of exosomes from microglia cells
Krabbe et al., 2012; Etienne et al., 2019	5-HTR, 5-HT2B, LPS, TNF-α, IL-6	Serotonin promotes microglia-induced targeted motility, but attenuates phagocytosis activity
Kolodziejczak et al., 2015	5-HT2B	Serotonin–microglia neurotransmission in development
Béchade et al., 2021	*5-ht2b*	In the lack of *5-ht2b*, overexpression of cytokines and prolonged neuroinflammation
de las Casas-Engel et al., 2013	5-HT7	Microglia-mediated serotonin neurotransmission to maintaining anti-inflammatory state
Mahé et al., 2005; Wixey et al., 2018	5-HT7	Present on human microglial MC-3 cells
Quintero-Villegas and Valdés-Ferrer, 2019	5-HT7, IL-6, AD	Promotes synaptogenesis and inflammatory priming *via* IL-6
Lim et al., 2009	FLX	Diminished microglia activation in ischemia
Liu et al., 2011	FLX, TNF-α, IL-6	Reduction in TNF-α and IL-6 secretion, *in vitro*
Jin et al., 2009	FLX, TNF-α, IL-1β	Fluoxetine-induced neuroprotection in the dentate gyrus following kainate-mediated neuronal cell death
Dhami et al., 2013	FLX, TNF-α, IL-1β	Reduction in the release of pro-inflammatory cytokines, and glutamate, *in vitro*
Alboni et al., 2016	FLX, TNF-α, IL-1β Iba-1, CD11b	Treatment on microglia activation and cytokine release differs depending on environmental conditions
MacGillivray et al., 2011	FLX, SERT, CD11b	Inhibition of SERT increases CD11b expression accompanied by loss of dopaminergic neurons
Zimniak et al., 2020	FLX	Attenuates symptoms in COVID-19 patients

*5-HT, 5-hydroxytryptamine; AD, Alzheimer’s disease; ATP, adenosine triphosphate; B2R, bradykinin receptor 2; BDNF, brain-derived neurotrophic factor and its receptor TrkB (tropomyosin-related kinase receptor B); BrdU, bromodeoxyuridine (cell proliferation marker), microglia marker CD11b Integrin αM, and Iba-1 (Ionized calcium binding adaptor molecule 1); ENR, enriched environment; FLX, fluoxetine; Interleukins, IL-1 to IL-18; LPS, lipopolysaccharide; SERT, serotonin transporter; TNF-α, tumor necrosis factor; Tph2, tryptophan hydroxylase 2; VMAT, vesicular monoamine transporter.*

### Microglia Function in Neuroplasticity—BDNF Signaling and Physiologic Stimuli

**FIGURE 1:**
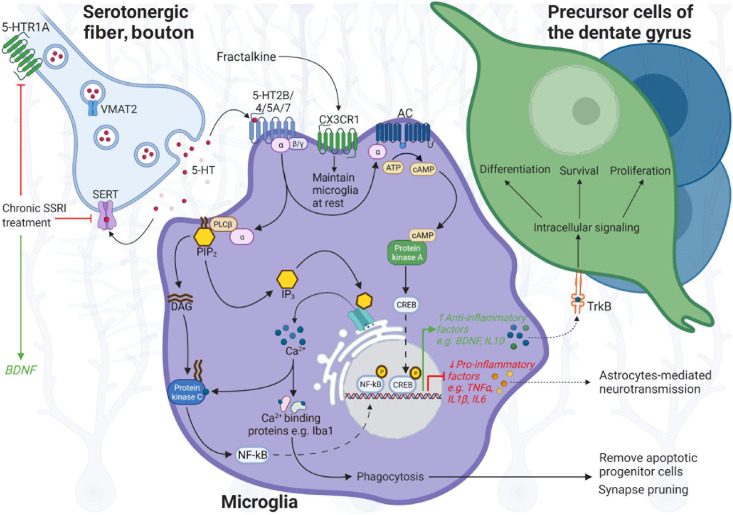
Illustration of microglia function in neuroplasticity of the hippocampus. In close proximity to precursor cells and neurons in the dentate gyrus, resting microglia control the neuronal cell pool by removal of apoptotic progenitor cells and synapse pruning, regulate synaptic plasticity and neural network excitability *via* ATP, and the release of TNF-α and BDNF, and respond to serotonin (5-HT) neurotransmission. Microglia express 5-HT receptors, most prominently 5-HT2B in response to serotonin, and CX3CR1 in response to neuronal fractalkine/CX3CL1 signaling that allows surveillance of the niche, and communication with neurons to maintain homeostasis. In particular, serotonin neurotransmission can direct microglia function toward neuroprotection or permit the response to inflammation. Dense tracts of serotonergic fibers terminate in the hippocampus. Upon receptor binding, 5-HT2B, coupled to Gαq/G11 protein, activates phospholipase C (PLC), which hydrolyzes phosphatidylinositol-4,5-bisphosphonate (PIP2), and mediates cellular effects through increasing levels of inositol triphosphate (IP3) and diacylglycerol (DAG). IP3 promotes Ca2^+^ excretion from endoplasmic reticulum, which activates Iba-1 involved in motility and phagocytosis activity of microglia, and is affected by 5-HT2B. IP3-induced Ca2^+^ release can also stimulate phospholipase C (PLC) (likewise *via* DAG), activating nuclear factor kappa beta (NF-kB) and in turn controls the expression of pro-inflammatory genes, e.g., TNF-α, IL-1β, and IL-6. Activated by 5-HT4 and 5-HT7 coupled to Gαs, the enzyme adenylate cyclase (AC) synthesizes the second messenger elevating cyclic AMP (cAMP) from ATP that activates protein kinase A (PKA); cAMP response element-binding protein (CREB) then controls transcription of genes involved in the anti-inflammatory response, BDNF or IL-10, exerting effects through their receptors, TrkB and IL-10R, located on precursor cells (TrkB) and neurons. 5-HT5A interactions with Gαi protein inhibit AC and downstream cascades. Upon harmful stimuli, microglia secrete pro-inflammatory cytokines, TNF-α, IL-1β, and IL-6, and actively remove cell debris. In prolonged neuroinflammation, microglia–neuron communication is altered, leading to neurodegeneration and cognitive deficits. In response to SSRIs, targeting SERT and presynaptic 5-HT1A auto-receptors on serotonergic neurons, 5-HT availability is enhanced in the synaptic cleft, which may also modulate BDNF levels. Increased release of pro-inflammatory cytokines may be counter-balanced by increased 5-HT levels upon SERT inhibition through fluoxetine—having anti-inflammatory properties. 5-HT, 5-hydroxytryptamine; BDNF, brain-derived neurotrophic factor and its receptor TrkB (tropomyosin-related kinase receptor B); Iba-1, ionized calcium binding adaptor molecule 1; SERT, serotonin transporter; TNF, tumor necrosis factor; VMAT2, vesicular monoamine transporter 2. *BioRender* was used to build the image.

Neuroplasticity in the adult hippocampus enables its structure to adapt to environmental challenges and novel experiences by rewiring upon learning and to respond to trauma or injury. Specifically, the discovery that new neurons are continuously generated has stirred hope for new therapeutic strategies to improve cognitive function and to treat neurodegenerative disorders. Microglia contribute to adult neurogenesis and memory formation (Gemma and Bachstetter, 2013). In close proximity to neurons and dendritic spines, microglia control synapse connectivity *via* secretion of TNF-α and adenosine triphosphate (ATP) that in turn promote astrocyte-mediated neurotransmission (Figure 1; Pascual et al., 2012). Activation of the ATP receptor subtype P2X4 drives BDNF release from microglia, which might display a central pathway in microglia–neuron signaling (Trang et al., 2011). BDNF is crucially involved in neuronal maturation and neurotransmission *via* binding to tropomyosin-related kinase receptor B (TrkB) located on neurons (Mattson, 2008). Microglial release of BDNF directly affects nearby synapse connectivity and promotes neuronal TrkB phosphorylation that enhances microglia–neuron interplay in learning (Parkhurst et al., 2013). BDNF released by activated microglia alters neuronal excitability by causing synaptic disinhibition (Ferrini and De Koninck, 2013). In their major role in synaptic pruning, microglia actively engulf and remove dysfunctional synapses from neuronal cell bodies in the uninjured brain (Paolicelli et al., 2011). In vicinity to NSCs in the subgranular zone, they remove apoptotic progenitor cells within the first days of cell birth (Sierra et al., 2010) through the phagocytosis secretome (Diaz-Aparicio et al., 2020), thereby balancing synaptogenesis and cell death.

Novel experiences and external stimuli influence NSC/progenitor behavior, and activity-dependent changes in neuroplasticity occur, including a robust increase in precursor cell proliferation upon running (van Praag et al., 1999; Kronenberg et al., 2003) and cell survival upon exposure to an enriched environment (ENR) (Kempermann and Gage, 1999). The neurogenic regulatory effect of running is mediated through central serotonin (Klempin et al., 2013), with circulatory factors, i.e., the angiotensin-converting enzyme 2 (Klempin et al., 2018) or cathepsin B released by skeletal muscle (Moon et al., 2016) contributing to increased precursor proliferation. Physical exercise increases microgliosis in the dentate gyrus of wild-type mice that is further enhanced in the absence of brain serotonin (Klempin et al., 2013), or in the lack of bradykinin B2 receptor (Wasinski et al., 2018). Physical activity increases the number of newborn microglia in the adult mouse cortex (Ehninger and Kempermann, 2003), while a reduction was observed in the adult amygdala upon running and ENR (Ehninger et al., 2011). While Iba-1 expression is reduced up to 2 months in ENR, long-term ENR conditions enhance microgliosis in adult hippocampus and amygdala accompanied by hypertrophied and ramified microglia morphology (Ali et al., 2019). Microglia proliferation and morphological transformation are characteristics of the vigilant phenotype that allows rapid adaptation to a demanding microenvironment. Thereby, cellular physiology including Ca2^+^ signaling and highly branched processes supports the sensor ability, while an amoeboid shape with dynamic extensions facilitates cellular locomotion toward the site of neural damage and factor release (Nayak et al., 2014). As a result of positive stimuli, physical exercise, and ENR, altered microglia phenotypes display neuroprotective functions. In ENR, microglia morphology in adult rodent brain shows increased complexity in CA3 (de Sousa et al., 2015) but decreased diversity in the molecular layer (de Oliveira et al., 2020). In animal models for several diseases, physical activity induces anti-inflammatory effects revealed by decreased microglia activation and Iba-1/CD11b expression, a ramified morphology, or normalization in synaptic density in CA3 (Andoh and Koyama, 2020). Exercise also strongly enhances BDNF signaling in mammals that in turn exerts positive cognitive effects (Johnson et al., 2003; Szuhany et al., 2015; Moon et al., 2016). Together, these studies show that microglia contribute to neuroplasticity and synaptic rewiring in the adult hippocampus and respond to physiologic stimuli that could ameliorate from pathologies.

### Microglia Function in Neuroinflammation and Neurodegeneration

Neuroplasticity of the adult brain can also be negatively regulated, inducing structural changes and impaired neurogenesis as has been observed in stress-related events (Tuchina et al., 2018), and is associated with age-related cognitive decline and neurodegenerative and psychiatric disorders in humans. Upon inflammation or following infection, an acute immune response comprises the release of pro-inflammatory cytokines and phagocytosis of damaged cells, mainly neurons (Suzumura, 2013). Notably, activation of endogenous microglial cells goes along with migration of blood-derived cells into the brain, such as in ischemia (Kronenberg et al., 2018b). Chronically activated microglia, increased cell density and hyper-ramified morphology (Dubbelaar et al., 2018), and the enhanced release of pro-inflammatory cytokines, e.g., TNF-α, IL-1β, and IL-18, are observed in response to stress, major depression, or AD, leading to disease progression and brain damage (Pickering and O’Connor, 2007). This “hyperactivation of the immune response” might be due to inefficiency in the phagocytosis phenotype of microglia. Indeed, similar characteristics are observed for aging, senescent myeloid cells, where an impaired immune response results from decreased process motility and chemotaxis (Goronzy and Weyand, 2013). Dysfunctional microglia might be a hallmark of late-stage AD development (Shen et al., 2018). In particular, microglial cells are in close proximity to β-amyloid plaques, one of the characteristics of disease progression; their processes engulf β-amyloid that leads to enhanced pro-inflammatory signaling, e.g., of TNF-α inducing cell death and the release of BDNF (Burbach et al., 2004; Floden et al., 2005). Although BDNF is anti-inflammatory and considered as a therapeutic target, increased BDNF signaling might negatively contribute to the aberrant axonal growth in AD in its role as modulator of neuronal and synapse maturation in healthy conditions. However, in an animal model of multiple sclerosis, BDNF promotes IL-10 that reduces clinical severity (Makar et al., 2009). Notably, increased peripheral levels of IL-1β, and of IL-1β and IL-6, but unchanged TNF-α, were reported in patients with AD or major depression, respectively (Ng et al., 2018).

Microglial release of inflammatory factors in the dentate gyrus differentially affects precursor cell proliferation, survival, and differentiation (Borsini et al., 2015). Secretion of anti-inflammatory IL-10 is involved in synaptic plasticity and long-term potentiation (Kelly et al., 2001; Lim et al., 2013) and counteracts the pro-inflammatory phenotype of chronically activated microglia (Cacci et al., 2008). In traumatic brain injury, repopulated microglia can adopt a phenotype that drives repair, specifically by promoting adult neurogenesis *via* soluble IL-6 receptor (Willis et al., 2020). Increased release of pro-inflammatory cytokines is observed upon decreased microglial CX3CR1 expression in response to fractalkine/CX3CL1 signaling deficiency, which results in a dramatic reduction in adult neurogenesis in chronic stress (Milior et al., 2016) and in AD (Bachstetter et al., 2015). Microglia *Cx3cr1* knockout mice display a transient early postnatal increase in synaptogenesis due to deficiency in synaptic pruning in the dentate gyrus (Paolicelli et al., 2011) that is independent of fractalkine signaling (Sellner et al., 2016), accompanied by reduced neuronal maturation of precursor cells and impaired learning and memory in the adult (Bolós et al., 2018). To model systemic inflammation *in vitro/in vivo*, bacterial lipopolysaccharide (LPS) is administered, resulting in increased microglia density and the release of pro-inflammatory factors IL-1β/IL-6 and TNF-α. A dose- and time-dependent decrease in proliferation and survival of precursor cells is observed *in vivo* in adult mouse and rat, respectively (Bastos et al., 2008; Fujioka and Akema, 2010), which is accompanied by a depressive-like state (Tang et al., 2016). Thereby, a dramatic reduction in newborn neurons is correlated with an increase in activated microglia (Ekdahl et al., 2003). *In vitro* experiments reveal that the number of precursor cells adopting a neuronal fate is significantly reduced when co-cultured with activated microglia expressing IL-6, while LPS added directly to precursor cells has no effect on neurogenesis (Monje et al., 2003). A single dose of LPS also significantly decreases the density of p-TrkB and BDNF protein in dentate gyrus and CA3 of young-adult male mice (Zhang et al., 2014). Likewise, an age-related decline in BDNF-TrkB signaling is accompanied by increased microglia activation (Wu et al., 2020). *Vice versa*, microglia activation, phenotypic transformation, and release of pro-inflammatory cytokines can be reduced by local supplement of BDNF *in vivo/in vitro* (Wu et al.) or TrkB agonist treatment *in vivo* (Zhang et al., 2014). BDNF induces sustained elevation of intracellular Ca2^+^ signaling and inhibits microglial production of nitric oxide (NO) (Mizoguchi et al., 2014).

### Serotonin–Microglia Interplay

Serotonin is the most widespread monoamine of the central nervous system, key signaling molecule in neuroplasticity of the hippocampus, and target in antidepressant therapy. Briefly, synthesized in neurons of the brain stem dorsal and median raphe nuclei (DRN, MRN) by the rate-limiting enzyme tryptophan hydroxylase 2, serotonin is packed into synaptic vesicles by the vesicular monoamine transporter (VMAT) 2, and upon release, re-uptake is regulated by the selective serotonin transporter, SERT (Gaspar et al., 2003). Earlier studies on brain serotonin–microglia interaction were done upon stimuli or pharmacological depletion of serotonergic neurons that results in increased microglia density, characterized by Iba-1 and CD11b expression, in DRN (Vetreno et al., 2017), or microgliosis in the subependymal layer of the subcommissural organ in adult rats (Stagaard et al., 1987). Neuroinflammation induced by systemic LPS reduces serotonin levels in the hippocampus that is accompanied by a depressive-like phenotype in rats (Carabelli et al., 2020). Chronic LPS activation only transiently increases microglia numbers and alters striatal and prefrontal serotonin signaling alongside depressive-like behavior (Krishna et al., 2016). Omega-3 administration leads to increased serotonin levels in the hippocampus and reverses the behavioral phenotype (Carabelli et al., 2020). Serotonin fiber pathways project into numerous brain areas and spinal cord. Target areas in the dentate gyrus, precursor cells and neurons, express various 5-HT receptors that control the response from efferent activity at different cell stages within the neuronal lineage (Brezun and Daszuta, 2000; Klempin et al., 2010). Recent studies establish that neurotransmitter receptors are not specific for neurons, but can be found on glial cells, and molecules are detected through diffuse non-synaptic transmission in the extracellular space (Pocock and Kettenmann, 2007). Serotonin, in particular, is released *via* boutons *en passant*, and ultrastructure imaging reveals brain serotonin–microglia interplay in the hippocampus with microglia processes in close proximity to serotonergic axons (Albertini et al., 2020). Seven groups (5-HT1–5-HT7) and their subtypes, with 5-HT3 as an exception, belong to the G-protein-coupled receptor family regulating different signaling pathways; almost all of them are expressed on distinct microglia subpopulations (Krabbe et al., 2012; Glebov et al., 2015).

Accumulating evidence attributes 5-HT2B receptor subtype an important role in microglia–neuron communication in rodent brain development (Kolodziejczak et al., 2015), and in microglia-mediated serotonin transmission. *In vitro* studies reveal enhanced microglia response to injury in acute mouse brain slices (Krabbe et al., 2012), and transiently boosted Ca2^+^ signaling in cultured resting microglia upon serotonin administration (Seifert et al., 2011). Specifically, activation of 5-HT2B leads to enhanced motility and oriented growths of microglial processes that is important in response to injury but decreases the phagocytosis activity (Krabbe et al., 2012; Etienne et al., 2019). In the lifelong absence of microglial *5-ht2b*, peripheral LPS injection causes cytokine overexpression and prolonged neuroinflammation *in vivo* that goes along with increased morphology transformation and hyper-ramification (Béchade et al., 2021). These studies suggest that serotonin is involved in the alterations of microglial phenotype as is known for peripheral macrophages (de las Casas-Engel et al., 2013). Together with 5-HT2, microglial expression of 5-HT4 is involved in the release of exosomes from microglia that is dependent on elevated cytosolic Ca2^+^ signaling (Glebov et al., 2015). Microglial secretion of cytokines modulated by serotonin neurotransmission might lead to maintenance of an anti-inflammatory state (de las Casas-Engel et al., 2013); indeed, secretion of pro-inflammatory factors TNF-α or IL-6 was unchanged during LPS stimulation in the presence of serotonin (Krabbe et al., 2012). Functional 5-HT7 receptors are present on human microglial MC-3 cells (Mahé et al., 2005; Wixey et al., 2018). 5-HT7 expressions on both neurons and microglia promote synaptogenesis and induce inflammatory priming *via* IL-6 production. In an AD animal model, reduced neurotoxicity of β-amyloid was observed in hippocampus upon administration of LP-211, a 5-HT7 agonist (Quintero-Villegas and Valdés-Ferrer, 2019). Collectively, these studies suggest serotonin’s role in keeping microglia in a resting, surveillance, and anti-inflammatory state.

### Serotonin–Microglia Interplay Upon Fluoxetine

Dysregulation of serotonin signaling is associated with neurogenic decline, age-related memory loss, and psychiatric disorders. SSRIs increase serotonin neurotransmission targeting SERT and specific 5-HT (auto-) receptors (Descarries and Riad, 2012) that leads to clinical improvement and is linked to a delayed increase in adult neurogenesis as shown in rodents (Malberg et al., 2000; Santarelli et al., 2003). BDNF has been implicated in the pro-neurogenic effects; SSRI-induced increases in serum BDNF have been detected in rodents (Nibuya et al., 1996), and similarly in depressed patients (Molendijk et al., 2011); however, BDNF protein in hippocampus of mice is not elevated (Petermann et al., 2020). Increasing evidence indicates that neurodegenerative diseases and psychiatric disorders are characterized by an immune-inflammatory state and that antidepressants not only improve mood but also possess anti-inflammatory properties. It is suggested that hyperactive microglia and increased pro-inflammatory cytokine levels result in elevated SERT expression as a consequence or interdependency to elevated serotonin levels. SSRIs target SERT function that in addition to inhibiting serotonin re-uptake might activate anti-inflammatory intracellular pathways (Walker, 2013): In LPS-induced primary microglia culture, incubation with serotonin significantly alters TNF-α production (Tynan et al., 2012). Likewise, pre-treatment with five different SSRIs, including fluoxetine, substantially inhibits IL-1β or IL-6 secretion (Liu et al., 2011) and microglial production of TNF-α and NO, with cyclic adenosine monophosphate signaling involved in the regulation of an anti-inflammatory response (Tynan et al., 2012). Co-cultured with cortical neurons, microglial release of the pro-inflammatory factors IL-1β, TNF-α, and glutamate was reduced upon fluoxetine and citalopram (Dhami et al., 2013). *In vivo* pre-treatment with fluoxetine or paroxetine attenuates LPS-induced increases in TNF-α serum levels (Ohgi et al., 2013). In models of neurodegenerative disease, fluoxetine administration reduces microglia activation in ischemia (Lim et al., 2009) and leads to recovery from kainate-induced cell death in the dentate gyrus (Jin et al., 2009). Depending on environmental challenges, cytokine release in hippocampus is differentially affected by fluoxetine resulting in increased pro-inflammatory IL-1β expression in ENR conditions, but decreased TNF-α production upon stress. However, microglia density and Iba-1/CD11b expression in hippocampus remain unchanged (Alboni et al., 2016). In contrast, in substantia nigra, SERT inhibition by fluoxetine increases microglia activation and CD11b immunoreactivity, leading to loss of dopaminergic neurons (MacGillivray et al., 2011). Together, microglia activity and release of cytokines can be modulated by serotonin neurotransmission, e.g., SERT-mediated clearance of released serotonin upon fluoxetine (Robson et al., 2017) and altered intrinsic cellular signaling. However, whether SERT is expressed on microglia lacks evidence.

An overreaction of the immune system, a “cytokine storm” (Ragab et al., 2020) is also associated with the pathophysiology following SARS-CoV-2 infection that might contribute to long-term neurological impairments. Preliminary results reveal that fluoxetine treatment specifically decreases viral protein expression in COVID-19 patients (Zimniak et al., 2020). Thus, SSRI treatment with anti-inflammatory effects given early might prevent both severe progression of the disease and chronic despair.

## Discussion

Over the past few years, it has become apparent that endogenous microglia of the adult brain take part in neuroplasticity of the hippocampus by controlling the neuronal cell pool, regulating synaptic plasticity in learning *via* release of TNF-α and BDNF, and responding to physical exercise (Table 1). Resting microglia express 5-HT2B and CX3CR1, constantly survey the niche’s microenvironment, and communicate with neurons to maintain homeostasis. In particular, serotonin neurotransmission can direct microglia function toward neuroprotection, or permit the response to inflammation (Pocock and Kettenmann, 2007). Upon harmful stimuli, microglia perform an innate immune response; secrete pro-inflammatory cytokines TNF-α, IL-1β, and IL-6; and actively remove cell debris, similar to peripheral macrophages. When toxic molecules are removed from the nervous tissue, microglia become “alternatively activated”, change their phenotype to anti-inflammatory (IL-10), and start restoring homeostasis (Lobo-Silva et al., 2016). However, in chronic diseases, neuron–microglia communication is somewhat altered, causing a prolonged inflammatory state, leading to impaired chemotaxis and phagocytosis. Hyperactivation of the immune response also impairs survival and differentiation of progenitor cells, which, together with impaired serotonin and BDNF signaling, are characteristics of major depression. With SSRIs such as fluoxetine targeting both signaling pathways and, in addition, enabling an anti-inflammatory response, microglia might display an add-on therapeutic target to improve psychiatric disorders, cognitive decline, or viral-induced neurological deficits. Nonetheless, considering the various factors involved and the vast heterogeneity of human microglial cells (Böttcher et al., 2019), there is a long road ahead.

## Author Contributions

AT, OT, and FK have equally contributed to designed and wrote the manuscript. All authors contributed to the article and approved the submitted version.

## Conflict of Interest

The authors declare that the research was conducted in the absence of any commercial or financial relationships that could be construed as a potential conflict of interest.
